# Editorial: Recent developments in artificial intelligence and radiomics

**DOI:** 10.3389/fmed.2026.1825215

**Published:** 2026-03-20

**Authors:** Luigi Manco, Salvatore Annunziata, Luca Urso

**Affiliations:** 1 Medical Physics Unit, University Hospital of Ferrara, Ferrara, Italy; 2 Nuclear Medicine Unit, Fondazione Policlinico Universitario A. Gemelli IRCCS, Rome, Italy; 3 Department of Translational Medicine, University of Ferrara, Ferrara, Italy

**Keywords:** AI, medical imaging, nuclear medicine, radiology, radiomics

Since the landmark definition of radiomics by Lambin in 2012, the discipline has transcended its initial role as a niche computational tool to become a cornerstone of modern imaging (1). We are currently witnessing a paradigm shift: the transition from subjective, qualitative image interpretation to a high-throughput, quantitative extraction of “hidden” data. The synergy between radiomics and Artificial Intelligence (AI) is no longer just a research promise; it is the engine driving precision medicine toward clinical reality. However, as this field matures, the “reproducibility crisis”—arising from a lack of standardization and multicenter generalizability—remains the crucial milestone for achieving full-scale clinical integration.

This Research Topic, *Recent developments in artificial intelligence and radiomics*, features 12 studies that show the new directions of this field. Expanding beyond the traditional oncologic focus, these works cover a wide range of medical areas: from modern oncology to orthopedics and various non-cancer conditions. Collectively, they demonstratehow AI (both deep learning—DL—and machine learning—ML) and radiomics can improve diagnostic accuracy, accelerate workflows, and help clinicians make better clinical decisions across different specialties (2).

In the oncological field, radiomics and AI are no longer “emerging tools” but the fault line along which oncologic imaging is splitting into past and future (3). What these four papers (Deng et al.; Lyu et al.; Wang, Wang, Wang et al.; Wang, Wang, Li et al.) collectively expose is a discipline that has outgrown its exploratory phase: the field is now seeking methodological maturity, and—crucially—clinical ambition. The bibliometric analyses make this undeniable (Deng et al.; Lyu et al.). Radiomics is expanding at a pace that traditional imaging research has never seen, shifting from handcrafted features to DL, from descriptive studies to clinically consequential prediction models. The dominant themes—survival prediction, metastatic risk, lymph-node stratification, AI-driven inference—signal a community no longer content with descriptive imaging studies but intent on building tools that will matter at the bedside.

At the same time, the application papers demonstrate the advanced integration of this technology into real-world complexity. Dosiomic predicting immunotherapy—era pneumonitis with AUCs approaching 0.94 is not incremental progress—it is a direct challenge to the dominance of DVH-based toxicity models (Wang, Wang, Wang et al.). In neuro-oncology, the AI systems described—nnU-Net variants, ensemble detectors, recurrent architectures—have evolved into formidable clinical instruments. They consistently match or exceed human precision in segmentation, accelerate reading times, and begin to tackle the holy grail of brain metastasis management: reliable differential diagnosis and prognostic modeling (Wang, Wang, Li et al.). The message is unmistakable: AI is not just reading images; it is extracting biology from them and decoding the underlying genomic alterations (4).

In the domain of orthopedic diseases, three studies investigated the potential of these novel tools applied to medical imaging. Kou et al. present a DL and radiomics–based system for the early diagnosis of hip synovitis in juvenile idiopathic arthritis (JIA), demonstrating how AI models can detect subtle ultrasound features often missed by the human eye. Their YOLO-JIA framework combines automated segmentation and radiomic classification to achieve impressive diagnostic accuracy (AUC 0.88 on internal and 0.81 on external validation), offering a valuable tool for early intervention in pediatric rheumatology. Liu, Chen et al. demonstrate the practical utility of an AI-assisted iterative reconstruction algorithm (AIIA) applied to knee MRI. Their study highlights how AI-powered post-processing can substantially reduce scan time—achieving a reduction of up to 50% in specific sequences—without compromising signal-to-noise ratio or diagnostic performance. By maintaining high image quality scores (comparable to standard-of-care protocols) while significantly shortening the acquisition window, this work proves how AI can enhance both workflow efficiency and patient comfort, successfully bridging the gap between technological innovation and clinical practicality. Finally, Xiang et al. introduce *HASA-ResUNet*, an innovative architecture that enhances the classic U-Net for knee MRI segmentation. By integrating hierarchical (HFEF) and atrous (ASA) attention modules, the model implements a multi-scale strategy that can effectively address the challenges of class imbalance in knee MRI images, improving the model's overall segmentation performance. This approach represents a crucial step forward for robust and automated musculoskeletal image analysis, particularly for detecting small and complex anatomical structures.

Moreover, several non-oncological conditions are poised to significantly benefit from radiomics and AI in the near future. A significant contribution, by Liu, Song et al. explores the differentiation of medullary sponge kidney from common nephrolithiasis. By fusing handcrafted radiomics with DL features, the authors achieved near-perfect diagnostic accuracy, with an AUC of 0.96 for their DL radiomics signature. This hybrid approach underscores the transformative potential of integrated models in solving complex diagnostic dilemmas in urology and improving preoperative decision-making.

Within the cardiovascular Diagnostics and Coronary Artery Disease (CAD), the study by Lo Iacono et al. addresses the challenge of automated coronary stenosis grading. The novelty of their research lies in a hybrid approach that combines 2D radiomic features with latent features extracted via Autoencoders (AE) from Coronary Computed Tomography Angiography (CCTA) images. Utilizing a dataset of 2,548 multiplanar reconstructed (MPR) images, the researchers implemented a cascade pipeline to classify patients into no-CAD, non-obstructive, or obstructive CAD. The combined model significantly outperformed single-method approaches, achieving a balanced accuracy of 0.91 and a specificity of 0.94. This suggests that the synergy between traditional radiomics and DL features is essential for robust coronary screening.

Zheng et al. investigated the application of MRI-based radiomics to predict neurological recovery following surgical decompression for Thoracic Spinal Stenosis (TSS)—a condition where traditional clinical markers often fail to provide accurate prognostic insights. In a retrospective study of 106 patients, radiomic features were extracted from T2 axial MRI scans and refined through rigorous selection processes (including LASSO and mRMR). The integrated radiomics-clinical model achieved a test-set AUC of 0.867, outperforming purely clinical models. The findings advocate for radiomics as an objective tool to support individualized surgical decision-making.

The multicenter research conducted by Xu et al. focused on monitoring liver iron burden in TM patients within 2 years of receiving a hematopoietic stem cell transplant (HSCT). The study analyzed 3.0T and 1.5T MRI images from 360 patients across two medical centers to evaluate the predictive efficacy of radiomics. Despite variations in hardware and parameters, the models (specifically T1-based) demonstrated exceptional performance, with AUCs reaching 0.942 in training sets. The study highlights the feasibility of constructing center-specific MRI models to track long-term post-transplant prognosis.

Finally, Feng et al. introduced Habitat Imaging to identify no-infectious acute exacerbations of COPD (AECOPD) using CT scans. The whole lung was segmented into three distinct “habitats” (emphysema-associated, bronchovascular, and parenchyma) using K-means clustering. The “habitat-total” model demonstrated strong diagnostic efficacy, with an AUC of 0.897 in the training cohort. Multivariate analysis identified the habitat score and GOLD stage as independent predictors, providing a potential biomarker for quantifying lung heterogeneity.

These studies collectively demonstrate that across diverse pathologies—from oncologic to orthopedic to miscellaneous conditions—the integration of radiomics and AI tools provides a level of detail invisible to the naked eye. The shift toward “habitat” analysis and hybrid DL models represents the next frontier in standardized, precision medicine.

Across these works, a shared tension emerges: science is advancing faster than the clinical infrastructure that should receive it. Standardization, reproducibility, multicenter validation, and workflow integration remain the critical areas for immediate development (3). But the trajectory is clear: radiomics and AI are positioning themselves not as adjuncts to imaging but as the computational backbone of tomorrow's precision medicine.
